# Efficacy and safety of transarterial chemoembolization with CalliSpheres® Microspheres in head and neck cancer

**DOI:** 10.3389/fsurg.2022.938305

**Published:** 2022-08-25

**Authors:** Fei Gao, Jinqi Gao, Kuiyang Wang, Lei Song

**Affiliations:** Intervention Treatment Department, The Second Hospital of Dalian Medical University, Dalian, China

**Keywords:** transarterial chemoembolization with CalliSpheres® Microspheres, head and neck cancer, treatment response, survival, safety profile

## Abstract

**Objective:**

Transarterial chemoembolization with CalliSpheres® Microspheres (CSM-TACE) presents favorable efficacy and tolerable safety in several cancers, while its application in head and neck cancer (HNC) is unclear. Thus, the current pilot study aims to evaluate the efficacy and safety of CSM-TACE in treating HNC.

**Methods:**

A total of 15 HNC patients receiving CSM-TACE at the Second Affiliated Hospital of Dalian Medical University from March 2017 to December 2021 were enrolled in this study. Moreover, treatment information, treatment response, progression-free survival (PFS), overall survival (OS), changes in liver and renal function indices, and adverse events were recorded.

**Results:**

There were nine patients receiving CSM-TACE as first-line treatment and six patients receiving CSM-TACE as second- or above-line treatment; meanwhile, there were seven, seven, and one patient undergoing one time, two times, and three times of CSM-TACE, respectively. Furthermore, the objective response rate (ORR) and the disease control rate (DCR) were 60.0% and 100%, respectively, at the first month; meanwhile, the ORR and the DCR were 53.3% and 73.3%, respectively, at the second month. Moreover, the 1-year PFS rate was 34.1%, and the 1-year OS rate was 38.9%. Additionally, no change in liver function indices (namely, total protein, albumin, total bilirubin, alanine aminotransferase, and aspartate aminotransferase) or in renal function indices (namely, creatinine and blood urea nitrogen) was found before and 1 month after treatment (all *P *> 0.05). Meanwhile, no severe adverse events were found during and after CSM-TACE.

**Conclusion:**

CSM-TACE illustrates favorable treatment response and survival benefits as well as a tolerable safety profile in HNC patients.

## Introduction

Head and neck cancer (HNC) affects the anatomical regions of the head and neck, such as oral and nasal cavity, and larynx (1, 2). It is viewed as one of the most common cancers worldwide, with the main risk factors being consumption of tobacco and alcohol as well as human papillomavirus infection (1, 3, 4). Meanwhile, HNC causes approximately 500,000 deaths annually, which accounts for nearly 3% of all cancerous deaths globally (1, 5). Over the past few decades, great advancements have been made in the treatment of unresectable HNC (radiation therapy, chemotherapy, immunotherapy, etc.); however, efficacy is limited and the safety profile is unfavorable (1, 2, 6–8). Considering that HNC causes a huge global burden on healthcare systems, the exploration of effective and safe treatment to improve the management of HNC has become the need of the hour (4, 9).

Conventional transarterial chemoembolization (cTACE) is able to kill tumor cells by embolizing the tumor blood–feeding artery and releasing chemotherapy drugs, and it has been widely applied in several cancers (10–12). For instance, cTACE provides good survival benefits and possesses a favorable safety profile in hepatocellular carcinoma (10); meanwhile, it has also been reported that cTACE elicits a favorable treatment response from lung cancer patients (11). Currently, drug-eluting beads TACE (DEB-TACE) is being proposed to achieve superior survival benefits in several cancers compared with cTACE, among which CalliSpheres® Microspheres (CSM) (the first microsphere product independently developed in China) can sustainedly and steadily release antitumor drugs in the tumor at high concentrations with favorable embolization efficacy (13–15). Moreover, past studies have proposed that CSM-TACE is effective and safe in treating several cancers (15–17), while its efficacy and safety in HNC are unclear.

Therefore, the current pilot study aims to explore the efficacy and safety of CSM-TACE in treating HNC patients.

## Methods

### Patients

This study consecutively recruited 15 HNC patients who were treated in the Second Affiliated Hospital of Dalian Medical University from March 2017 to April 2021. The inclusion criteria were: (i) those diagnosed as HNC by pathology, cytology, and imaging examinations; (ii) aged 18 years or older; (iii) about to receive CSM-TACE treatment based on clinical status and willingness; and (iv) available to be followed up regularly. The exclusion criteria were: (i) those allergic to the materials used in the study; (ii) having severe and uncorrectable coagulation abnormalities; (iii) complications with severe infections or other cancers; and (iv) women who had a positive pregnancy test or were breastfeeding. The informed consent forms were signed by the patients themselves. The study was approved by the Ethics Committee.

### Data Collection

After recruitment and examination, the following clinical characteristics of the patients were recorded: (i) *demographic characteristics*: age, gender, height, weight, smoke status, and drink status; (ii) *chronic comorbidities*: hypertension and diabetes; (iii) *disease characteristics*: histological classification, tumor–node–metastasis (TNM) stage, number of tumors, location of tumor, and the largest tumor size; (iv) *blood routine examination*: white blood cell (WBC), red blood cell (RBC), absolute neutrophil count (ANC), monocyte (MONO), and platelet (PLT); (v) *coagulation function indices*: prothrombin time (PT), international normalized ratio (INR), and activated partial thromboplastin time (APTT); (vi) *previous treatment history*: history of surgery resection, history of chemotherapy, history of radiotherapy, and history of immunotherapy or targeted therapy; and (vii) *current treatment*: treatment line and times of CSM-TACE.

### Treatment

In this study, CalliSpheres® Microspheres (a diameter of 100–300 μm; Jiangsu Hengrui Medicine Co, Ltd., Jiangsu, China) loaded with oxaliplatin (80 mg), bevacizumab (100 mg), epirubicin (40 mg), or cisplatin (40–60 mg) were used as the chemoembolization reagent. CSM-TACE operations were performed in the digital subtraction angiography room. The detailed CSM-TACE procedures are as follows: after routine disinfection, the femoral artery was punctured by the Seldinger technique, and then angiography for the external carotid artery was performed to detect the tumor-supplying vessel. Next, superselective catheterization was performed according to the different areas in which lesions were located, such as the maxillary artery, lingual artery, ascending pharyngeal artery, and superficial temporal artery. Sequentially, the chemoembolization reagent was slowly injected through a microcatheter to the tumor-supplying vessel. The absorbable gelatin sponge microspheres were applied as complementary embolization materials if needed. The endpoint of embolization was stagnation of blood flow in the tumor-supplying vessel. Based on the treatment response at approximately 1 month after CSM-TACE, some patients received treatment to improve efficacy: six patients received cTACE or CSM-TACE; seven received arterial infusion chemotherapy.

### Efficacy Evaluation

The treatment response was evaluated using computed tomography examination according to the modified response evaluation criteria in solid tumors at 1 month and 2 months after CSM-TACE operation (18), including complete response (CR), partial response (PR), stable disease (SD), and progressive disease (PD). The proportion of patients achieving CR and PR was defined as the objective response rate (ORR), and the proportion of patients achieving CR, PR, and SD was defined as the disease control rate (DCR). In addition, all patients were followed up regularly, and the median follow-up period was 5.2 months. The last date of follow-up was December 20, 2021. Based on the follow-up, the progression-free survival (PFS) rate and the overall survival (OS) rate were calculated. PFS was defined as the duration from the CSM-TACE operation to disease progression or the patient's death; OS was defined as the duration from the CSM-TACE operation to the patient's death.

### Safety Evaluation

Liver function indices and renal function indices were used to evaluate the safety of treatment, which was assessed before treatment and 1 month after treatment. The liver function indices included total protein (TP), albumin (ALB), total bilirubin (TBIL), alanine aminotransferase (ALT), and aspartate aminotransferase (AST). The renal function indices included creatinine (Cr) and blood urea nitrogen (BUN). In addition, adverse events during and after CSM-TACE were also recorded, such as allergy, pain, fever, vomiting, and spinal cord injury.

### Statistics

All statistical analyses were conducted using SPSS Software, version 21.0 (IBM, San Jose, CA, USA), and all graphs were plotted using GraphPad Prism Software, version 6.01 (GraphPad Software Inc., San Diego, CA, USA). Data were expressed as the number of patients (%), mean ± standard deviation, or median (interquartile range). Kaplan–Meier curves were applied to show the PFS and OS of patients. Comparisons of biochemical indices before and after CSM-TACE treatment were analyzed using the Wilcoxon signed-rank test. The significance level of statistics was set as 0.05.

## Results

### Clinical Characteristics and Treatment Information

Among the 15 HNC patients, the mean age was 60.8 ± 10.6 years; out of these 15, there were 3 (20.0%) females and 12 (80.0%) males. With regard to histological classification, 12 (80.0%) patients had squamous cell carcinoma, 1 (6.7%) had adenocarcinoma, and 2 (13.3%) had other disease conditions. In terms of the TNM stage, there were four (26.7%) patients with stage II, four (26.7%) with stage III, and seven (46.7%) with stage IV. As far as the tumor location was concerned, five (33.3%) patients had tongue tumor, five (33.3%) had oral cavity tumor, two (13.3%) had neck tumor, one (6.7%) had cervical lymph node, one (6.7%) had throat tumor, and one (6.7%) had thyroid tumor; besides, the mean of the largest tumor size was 4.4 ± 2.6 cm. More clinical characteristics are detailed in Table 1.

**Table 1:** Clinical characteristics.

Items	HNC patients (*N* = 15)
Demographic characteristics	
Age (years), mean ± SD	60.8 ± 10.6
Gender, No. (%)	
Female	3 (20.0)
Male	12 (80.0)
Height (cm), mean ± SD	172.1 ± 7.9
Weight (kg), mean ± SD	61.9 ± 8.6
Smoke status, No. (%)	
Never	12 (80.0)
Former	3 (20.0)
Drink status, No. (%)	
Never	12 (80.0)
Former	3 (20.0)
Chronic comorbidities	
Hypertension, No. (%)	2 (13.3)
Diabetes, No. (%)	2 (13.3)
Disease characteristics	
Histological classification, No. (%)	
SCC	12 (80.0)
ADC	1 (6.7)
Others	2 (13.3)
TNM stage, No. (%)	
II	4 (26.7)
III	4 (26.7)
IV	7 (46.7)
Number of tumors, No. (%)	
Single	13 (86.7)
Multiple	2 (13.3)
Location of tumor, No. (%)	
Tongue	5 (33.3)
Oral cavity	5 (33.3)
Neck	2 (13.3)
Cervical lymph node	1 (6.7)
Throat	1 (6.7)
Thyroid	1 (6.7)
Largest tumor size (cm), mean ± SD	4.4 ± 2.6
Blood routine examination	
WBC (×10^9^/L), median (IQR)	6.3 (5.1–7.8)
RBC (×10^12^/L), median (IQR)	4.0 (2.9–4.5)
ANC (×10^9^/L), median (IQR)	4.8 (3.8–7.2)
MONO (×10^9^/L), median (IQR)	0.5 (0.3–1.0)
PLT (×10^12^/L), median (IQR)	220.0 (181.2–387.0)
Coagulation function indices	
PT (s), median (IQR)	13.1 (12.3–13.5)
INR, median (IQR)	1.0 (0.9–1.1)
APTT (s), median (IQR)	37.0 (34.1–37.9)

HNC, head and neck cancer; SD, standard deviation; SCC, squamous cell carcinoma; ADC, adenocarcinoma; TNM, tumor–node–metastasis; WBC, white blood cell; IQR, interquartile range; RBC, red blood cell; ANC, absolute neutrophil count; MONO, monocyte; PLT, platelet; PT, prothrombin time; INR, international normalized ratio; APTT, activated partial thromboplastin time.

With regard to treatment history, five (33.3%) patients were treated by chemotherapy, five (33.3%) received radiotherapy, and one (6.7%) underwent immunotherapy/targeted therapy. In terms of current CSM-TACE treatment, nine (60.0%) patients received first-line CSM-TACE and six (40.0%) received second- or above-line CSM-TACE; meanwhile, seven (46.7%), seven (46.7%), and one (6.7%) patient received one time, two times, and three times of CSM-TACE, respectively (Table 2).

**Table 2:** Treatment information.

Items	HNC patients (*N* = 15)
Previous treatment history	
History of chemotherapy, No. (%)	5 (33.3)
History of radiotherapy, No. (%)	5 (33.3)
History of immunotherapy/targeted therapy, No. (%)	1 (6.7)
Current CSM-TACE treatment	
Treatment lines, No. (%)	
First line	9 (60.0)
Second line or above	6 (40.0)
Times of CSM-TACE, No. (%)	
1 time	7 (46.7)
2 times	7 (46.7)
3 times	1 (6.7)

HNC, head and neck cancer; CSM-TACE, transarterial chemoembolization with CalliSpheres® microspheres.

### Treatment Response

After one month of treatment, five (33.3%) patients achieved CR, four (26.7%) achieved PR, six (40.0%) realized SD, and none (0.0%) had PD; meanwhile, the ORR and DCR were 60.0% and 100%, respectively. In addition, 3 patients lost follow-up after 2 months of treatment, so the treatment response was assessed among 12 patients. The data showed that after 2 months of treatment, four (26.7%), four (26.7%), three (20.0%), and one (6.7%) patient achieved CR, PR, SD, and PD, respectively; besides, the ORR and DCR were 53.3% and 73.3%, respectively (Table 3).

**Table 3:** Treatment response.

Items, No. (%)	One month after treatment (*N* = 15)	Two months after treatment^a^ (*N* = 12)
Overall response		
CR	5 (33.3)	4 (26.7)
PR	4 (26.7)	4 (26.7)
SD	6 (40.0)	3 (20.0)
PD	0 (0.0)	1 (6.7)
Missing	0 (0.0)	3 (20.0)
ORR (CR + PR)	9 (60.0)	8 (53.3)
DCR (CR + PR + SD)	15 (100.0)	11 (73.3)

CR, complete response; PR, partial response; SD, stable disease; PD, progressive disease; ORR, objective response rate; DCR, disease control rate.

^a^ Three patients were lost to follow-up at 2 months after treatment, so the treatment response at 2 months was assessed among 12 patients.

### Survival

**Figure 1:**
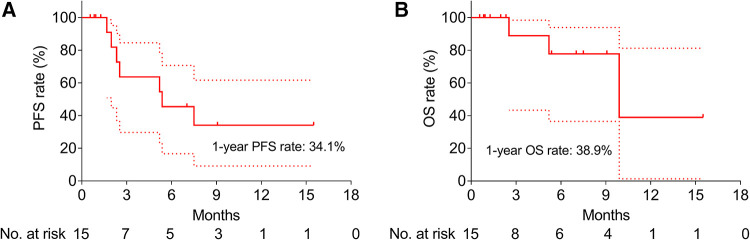
Survival of HNC patients after CSM-TACE. PFS (**A**) and OS (**B**) among HNC patients after CSM-TACE. PFS, progression-free survival; OS, overall survival; HNC, head and neck cancer; CSM-TACE, transarterial chemoembolization with CalliSpheres® Microspheres; dotted line, 95% confidence interval.

In order to evaluate the long-term efficacy of CSM-TACE among HNC patients, PFS and OS rates were calculated; the 1-year PFS rate was 34.1% (Figure 1A) and the 1-year OS rate was 38.9% (Figure 1B).

Moreover, PFS (*P *= 0.456) (Supplementary Figure 1A) and OS (*P *= 0.590) (Supplementary Figure 1B) did not vary between patients receiving CSM-TACE as first-line treatment and those receiving CSM-TACE as second-line or above-line treatment. Furthermore, no difference in PFS (*P *= 0.321) (Supplementary Figure 2A) and OS (*P *= 0.579) (Supplementary Figure 2B) was found in HNC patients having tumors in different locations. In addition, the key clinical characteristics and treatment outcomes of each patient are shown in Table 4 for a detailed presentation.

**Table 4:** Key characteristics and treatment outcomes of each patient.

No.	Age (years)	Gender	Histological classification	TNM stage	Tumor location	Times of CSM-TACE	Treatment response		Disease progression	Time to progression (months)	Death	OS (months)
							One month	Two months				
1	58	Male	SCC	III	Tongue	1	PR	CR	No	9.1	No	9.1
2	56	Male	Other	II	Oral cavity	2	CR	CR	No	4.8	Yes	5.2
3	64	Male	SCC	IV	Oral cavity	1	SD	Not assessed	No	1.3	No	1.3
4	48	Female	SCC	III	Tongue	3	PR	PR	Yes	2.3	No	2.3
5	54	Male	SCC	IV	Cervical lymph node	1	SD	SD	No	0.8	No	0.8
6	59	Male	SCC	IV	Throat	1	SD	PR	No	15.5	No	15.5
7	49	Male	SCC	III	Neck	1	CR	CR	No	2.4	Yes	2.5
8	88	Female	Other	IV	Thyroid	2	SD	PR	No	9.1	No	9.1
9	64	Female	SCC	II	Tongue	2	SD	PD	Yes	7.5	No	7.5
10	65	Male	SCC	II	Oral cavity	2	SD	Not assessed	No	0.9	No	0.9
11	71	Male	ADC	IV	Oral cavity	2	PR	PR	Yes	1.7	Yes	9.9
12	50	Male	SCC	IV	Oral cavity	1	CR	CR	No	7.0	No	7.0
13	53	Male	SCC	IV	Neck	2	PR	SD	Yes	0.6	No	0.6
14	72	Male	SCC	II	Tongue	2	CR	Not assessed	Yes	5.4	No	5.4
15	61	Male	SCC	III	Tongue	1	CR	SD	Yes	2.0	No	2.0

TNM, tumor–node–metastasis; CSM-TACE, transarterial chemoembolization with CalliSpheres® microspheres; OS, overall survival; SCC, squamous cell carcinoma; ADC, adenocarcinoma; PR, partial remission; SD, stable disease; CR, complete remission; PD, progressive disease.

### Liver, Renal Functions, and Adverse Events

No change in liver function indices (namely, TP, ALB, TBIL, ALT, and AST) and in renal function indices (namely, Cr and BUN) was found before and 1 month after treatment (all *P *> 0.05) (Table 5). In addition, there were no severe adverse events such as allergy, pain, fever, vomiting, and spinal cord injury.

**Table 5:** Biochemical indices.

Items, median (IQR)	Before treatment	One month after treatment	*P*-value
Liver function indices			
TP (g/L)	69.5 (62.2–74.7)	70.6 (66.2–74.6)	0.397
ALB (g/L)	37.1 (32.4–40.8)	38.0 (34.9–44.1)	0.510
TBIL (μmol/l)	10.0 (9.0–16.4)	10.1 (6.8–13.9)	0.272
ALT (U/L)	13.0 (10.5–18.4)	15.9 (11.6–22.3)	0.683
AST (U/L)	17.6 (14.0–21.7)	19.9 (17.3–24.5)	0.221
Renal function indices			
Cr (μmol/l)	69.7 (56.0–78.4)	73.2 (54.0–80.8)	0.638
BUN (mmol/l)	5.1 (4.3–6.3)	5.4 (3.6–6.3)	0.778

IQR, interquartile range; TP, total protein; ALB, albumin; TBIL, total bilirubin; ALT, alanine aminotransferase; AST, aspartate aminotransferase; Cr, creatinine; BUN, blood urea nitrogen.

## Discussion

Radiotherapy plays an extremely important role in serving as the first-line treatment of both early and locally advanced patients. According to the recommendations given by National Comprehensive Cancer Network (NCCN) guidelines and Chinese Society of Clinical Oncology (CSCO) guidelines, applying radiotherapy as an early postoperative adjuvant therapy has been considered as the primary and nonsurgical choice for locally advanced patients, while chemotherapy has been recommended for locally advanced or advanced patients (19, 20). However, treatment response is still unsatisfactory among HNC patients after the current treatment regimen (21–25). For instance, the ORR reaches 46% among HNC patients after radiotherapy. However, radiation causes postradiation damage to the irradiated area, including changes in skin properties, local vascular toughness, and local soft tissue fibrosis. Irreversible tissue damage is often a result of radiotherapy, which affects subsequent treatment (26). Furthermore, HNC patients undergoing chemotherapy achieve an ORR of 29.9% (25); moreover, it has been reported that HNC patients achieve an ORR of 33% after immunotherapy (21). Thus, the exploration of new types of treatment has become necessary.

In this study, the ORR and DCR were 60.0% and 100% after 1 month of CSM-TACE, and they were 53.3% and 73.3% after 2 months of CSM-TACE. Compared with HNC treatment recommended by various guidelines (including National Comprehensive Cancer Network Clinical Practice Guidelines and Chinese Society of Clinical Oncology diagnosis and treatment guidelines for head and neck cancer 2018), HNC patients receiving CSM-TACE realized elevated ORR, DCR, and PFS than those receiving chemotherapy and immunotherapy (6, 19, 20). The possible reasons for this might be that: (1) CSM could effectively embolize tumor blood supply vessels to induce tumor necrosis and meanwhile sustainably release antitumor drugs to maintain locoregional high concentrations, which resulted in satisfactory tumor treatment response (27); and (2) the different composition of patients might affect treatment response.

Until now, the prognosis of HNC patients is still unfavorable (1–3). It has been proposed that the median PFS is 3.6 months among HNC patients receiving chemotherapy (25); meanwhile, the OS rate is 16% among HNC patients receiving immunotherapy (28). In this study, the 1-year PFS rate was 34.1% and the 1-year OS rate was 38.9% after CSM-TACE, which were numerically prolonged than chemotherapy and immunotherapy (22, 28). The potential reasons for this might be that: (1) CSM-TACE served as a terminal embolization, which could block the blood supply of tumor lesions to the greatest extent possible; meanwhile, CSM-TACE could fully plug tumor target vessels and minimize tumor blood supply due to the small size of CalliSpheres® Microspheres (15); (2) CalliSpheres® Microspheres could release drugs slowly but continuously, and therefore, the local concentration of chemotherapy drugs in tumor lesions could be reached over a long period of time, which led to prolonged survival (17); and (3) CSM-TACE was characterized by a long period of effective time, and thus, it achieved favorable survival rates among HNC patients (29).

With regard to the safety profile of CSM-TACE, a previous study illustrated that liver function indices (namely, TP, TBIL, ALT, and AST) were similar among hepatocellular carcinoma patients before and after 1–3 months of CSM-TACE; meanwhile, the main adverse events included pain, fever, nausea, and vomiting, which were all manageable and tolerable (30). Another interesting research proposed that only mild pain and fever were observed among locally advanced breast cancer patients after CSM-TACE (16). In this study, we recorded changes in liver and renal function indices before and after treatment, as well as adverse events during and after treatment. Surprisingly, the data showed that no changes in the liver and renal function indices occurred before and after CSM-TACE; meanwhile, the postoperative adverse events were only mild and tolerable pain. Besides, there was no focal necrosis with abscess and surrounding tissue ischemia necrosis. The potential reason for this might be that CSM could directly release antitumor drugs into the target tumor, which consequently reduced the systemic toxicity; hence, the safety profile of CSM-TACE was favorable among HNC patients.

Compared with the HNC treatment recommended by the existing authority guidelines, HNC patients receiving CSM-TACE achieved better ORR, DCR, and PFS than those receiving conventional treatments (including chemotherapy and immunotherapy) (21, 26, 28). Furthermore, with regard to the postoperative adverse events among HNC patients receiving CSM-TACE, no grade III and IV adverse events were found but only mild and tolerable pain; besides, there was no focal necrosis among patients with abscess and surrounding tissue ischemia necrosis.

CSM-TACE was a relatively novel treatment method for HNC patients, which led to a limited sample size; thus, we enrolled patients having tumors in six different locations in the HNC area. In addition, four patients with stage II tumors had a surgical contraindication or were unwilling to receive surgery; thus, a decision to perform CSM-TACE on them depended on their willingness. Moreover, such a decision to be taken for all the included 15 patients was codetermined by patients and doctors; as far as the patients were concerned, they were willing to receive CSM-TACE as a first-choice treatment; the doctors , on their part, wanted to ensure that patients benefited from CSM-TACE. Additionally, the primary tumor for the included patients was different; thus, the loaded chemotherapeutic drug was different, while no difference in treatment efficacy was found in CSM-TACE loaded with different drugs.

However, there were several limitations in the present study: (1) this was an exploratory pilot study with a small sample size, and thus, studies with larger sample sizes could be conducted in the future; (2) this was a single-arm study, and thus, randomized controlled trials could be conducted to further explore the efficacy and safety of CSM-TACE in HNC patients; (3) the efficacy of CSM-TACE with different diameters of CSM in HNC patients could be investigated subsequently; and (4) the efficacy of DEB-TACE with other microspheres in HNC patients could be explored in the future.

In conclusion, CSM-TACE illustrates favorable treatment response and survival benefits, as well as a tolerable safety profile in HNC patients, which may provide a potential treatment choice for the management of HNC, while further validation by a larger sample size study is needed.

## Data Availability

The original contributions presented in the study are included in the article/Supplementary Material, further inquiries can be directed to the corresponding authors.
