# Supplementary figures and images for: Efficacy and safety of transarterial chemoembolization with CalliSpheres® Microspheres in head and neck cancer
Source: Front Surg. 2022 Aug 25;9:938305. doi: 10.3389/fsurg.2022.938305 (PMC9452835; doi:10.3389/fsurg.2022.938305)

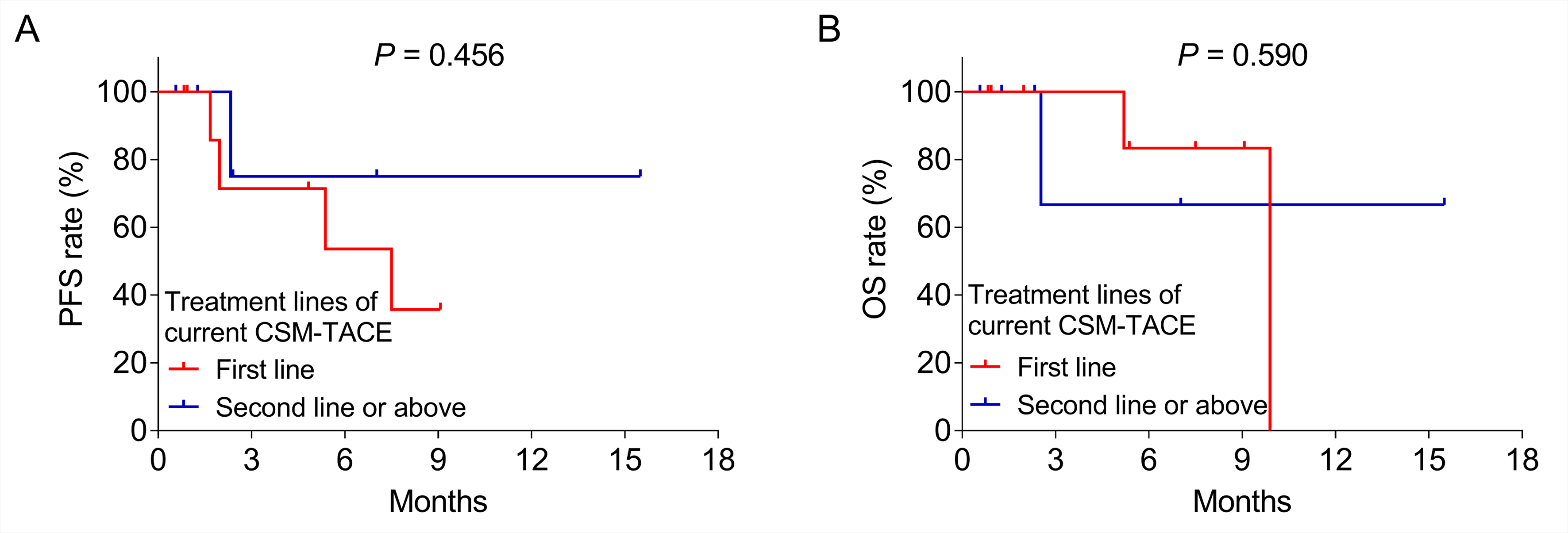

Supplement: Supplementary file 1 [file Image_1_v1.tif]

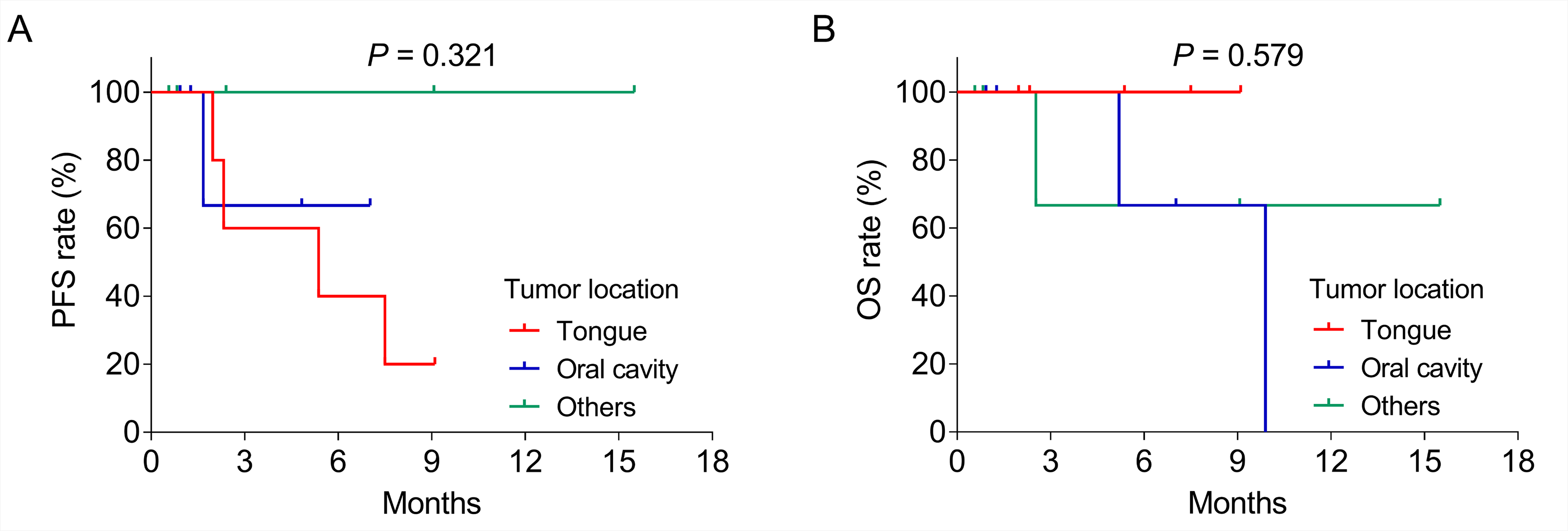

Supplement: Supplementary file 2 [file Image_2_v1.tif]
